# Process Evaluation of Food Game: A Gamified School-Based Intervention to Promote Healthier and More Sustainable Dietary Choices

**DOI:** 10.1007/s10935-023-00741-3

**Published:** 2023-08-06

**Authors:** Giovanni Aresi, Martina Giampaolo, Benedetta Chiavegatti, Elena Marta

**Affiliations:** 1 Psychology Department, Università Cattolica del Sacro Cuore, Largo Gemelli 1, Milano, 20123 Italy; 2 CERISVICO Research Centre on Community Development and Organizational Quality of Life, via Trieste 17, Brescia, 25121 Italy; 3 Unità Complessa Igiene Alimenti e Nutrizione, Azienda Tutela della Salute, Città Metropolitana di Milano, Italy

**Keywords:** Intervention, Mediterranean diet, Sustainability, Gamification, Process evaluation, Italy

## Abstract

Food Game is a gamified school-based intervention that aims to promote healthier dietary choices (i.e., adherence to the Mediterranean diet) and more sustainable behaviors among high school students in an urban area in Northern Italy. The program consists of an offline and online competition in which groups of students participate in peer-led activities to design and communicate products promoting health and sustainability. This study aimed to examine how the program works in practice, understand its mechanisms of change and assess any variation in student outcomes. A mixed methods process evaluation was conducted. Students completed a three-wave longitudinal survey and participated in focus groups. Program staff and teachers were also interviewed. Qualitative and quantitative data analyses indicate that Food Game?s gamification strategy was successful in engaging students, who felt stimulated by the game, its embedded competition and the self-organized group work. Although no significant change in adherence to the Mediterranean diet was found, pro-environmental behaviors, attitudes and perceived peer approval on healthy eating increased over time. Findings provide preliminary support for Food Game as an acceptable and engaging intervention though there is yet not sufficient evidence that it is also promotes healthier and more sustainable behaviors. The results contribute to the limited evidence base for offline gamified school-based interventions and offer insight into the applicability of the gamification approach as a key motivational strategy in other prevention settings and contexts. Suggestions on how to strengthen the program include dosage increase and addition of activities to promote change on relevant school and community factors.

**Supplementary Information:**

The online version contains supplementary material available at 10.1007/s10935-023-00741-3.

## Introduction

Lifestyle is a crucial factor in determining not only the health of people but also of the ecosystem. It has been widely acknowledged that unhealthy lifestyles are major risk factors for various chronic diseases (i.e., cardiovascular disease, diabetes and cancer) and premature death (WHO, 2014). From an environmental perspective, food consumption is one crucial aspect of lifestyle. There is evidence that food production, in particular of meat, has a large impact on the environment in terms of greenhouse gas emissions, land use and water footprint (Stehfest et al., 2009).

Therefore, shifts in dietary choices are viable opportunities to prevent disease and reduce environmental impacts, thus contributing to achieving the UN sustainable development goals (Willett et al., 2019). Accordingly, there is growing interest among researchers, stakeholders and decision-makers in developing and evaluating evidence-base policies and interventions to promote changes in the population’s dietary patterns.

## The Mediterranean Diet as a Model of Healthy and Sustainable Dietary Pattern

Italy and other countries in Southern Europe have long been associated with the Mediterranean diet (MedDiet) (Dernini & Berry, 2015; González-García et al., 2020). The MedDiet – declared an intangible cultural heritage of humanity by UNESCO in 2010 – is a traditional dietary pattern characterized by high intakes of vegetables, pulses, fruits, nuts, whole grain, fish and unsaturated fat and low intakes of red or processed meat. This diet is considered both healthy and sustainable due to its health and nutritional benefits, low environmental impacts and richness in biodiversity as well as other cultural and local economy positive returns (Dernini & Berry, 2015). A recent study showed an increasing trend in adherence to the MedDiet among adults and elderly across most socioeconomic subpopulations and countries in Europe (Alves & Perelman, 2022), though this trend appears to be reversed among young generations, more evidently among those of lower socio-economic status (Iaccarino Idelson et al., 2017). Lifestyle changes related to globalization and urbanization are considered to be responsible for such transition away from diets dominated by grains and vegetables towards diets rich in animal products as well as energy-dense and processed food among young people in Western countries (Hawkes et al., 2009) and, more recently, in countries like China (Yuan et al., 2019). Policies and interventions to tackle the abandonment of the MedDiet among youth in countries where this trend has been traditionally dominant can contribute to achieving better health and a more sustainable use of resources in these populations.

## Gamification in School-based Interventions

Habits related to lifestyle are learnt during childhood and adolescence and tend to persist throughout life. Therefore, childhood and adolescence represent a unique developmental window where behavior change interventions can have life-long consequences (Degenhardt et al., 2013; Smedbråten et al., 2022). Despite well-known challenges in schools’ capacity to sustain health interventions (e.g., staff motivation, organizational capacity) (Herlitz et al., 2020), these settings can make a difference in students’ health and represent an ideal setting for delivering any interventions as they can be easily implemented and evaluated and because the goal of providing a supportive healthy and sustainable environment for children aligns with schools’ core mission (Macnab et al., 2014).

It is well recognized that knowledge transmission is not enough to change lifestyles. A recent review demonstrated that, among the most effective dietary behavior change strategies, peer involvement including group discussions and practical activities ranked highest while provision of information ranked low (Calvert et al., 2019). The inclusion of peer-led learning activities within an intervention favors change because it provides opportunities for modelling positive behavior, promotes changes in social norms and creates a sense of belonging within a social group (Harden et al., 1999; Zha et al., 2019).

However, engaging young people in any activity is not easy and prevention is no exception. Gamification has been proposed as a strategy to induce engaging, positive psychological experiences and foster intrinsic motivation to participate and ultimately changing behavior. It refers to the inclusion of game elements to non-gaming contexts. Typical elements in gamification include goal setting, customized challenges, rewards and recognition, competition, cooperation and taking new roles. Gamification has been implemented in a variety of contexts, from exercise to employee engagement in the workplace (Hamari & Koivisto, 2015), though its application in health promotion is still an emerging trend and remains largely confined to apps and online settings (Edwards et al., 2016; Johnson et al., 2016).

This study describes results of a mixed methods process evaluation of Food Game, a gamified school-based intervention promoting healthier and more sustainable dietary choices (i.e., the Mediterranean diet) among high school students in an urban area in Northern Italy.

## The Food Game Program

Started in 2015, Food Game is a gamified school-based program––with an online component––that aims to increase the adoption of healthier and more sustainable behaviors (i.e., MedDiet and awareness of environmental issues) among high school students^1^. Food Game is implemented in the Milan metropolitan health district by the local health agency (ATS Città metropolitana di Milano, Lombardy region, Italy). The health district is an urban area that gathers 133 municipalities, including Italy’s second largest city, and a population of approximately 3.2 million in 2021. Out of this population, 75.000 people are between 15 and 19 years old and 11.9% are foreign-born. Food Game is aligned with the Lombardy region prevention plan. In the school context, this plans promotes a whole-school approach where other evidence-based health interventions, such as the Life Skills Training program, are delivered at scale (Velasco et al., 2015).

In the 2021/2022 edition, 16 schools were invited and 9 participated. Students attending the second and third year of high school (i.e., 14 to 15-year-olds) are the target with no further inclusion or exclusion criteria. Schools select a teacher tutor to support student teams and to facilitate communication with the health agency staff. While there is not a specific profile for teachers, generally those who are already responsible for health issues in the school, run physical activities or are class supervisors are most likely to be selected. Once selected, teacher tutors receive an induction to Food Game consisting of an individual meeting where the key features of the program are discussed along with what is expected from them. A group meeting is then organized with tutors from all schools. Tutors are also invited to watch a video on the program for further clarification. Once the program starts at the beginning of the school year, teachers get continuous support by the staff through e-mails or phone calls.

Food Game reflects the principles of peer-led learning (i.e., peer-led activities where students facilitate learning within a group) (Zha et al., 2019). In the program, students are encouraged to perform a number of peer-led activities in groups and gamification is used as the key motivational design. Students work in teams consisting of 20–30 people from the same class and compete with other teams, including from other schools. Team size reflects that of classes in the Italian school system. Teams have a list of 30 thematic challenges (e.g., organizing a fruit-day at school) to choose from (Table 1). Challenges identify a main goal but are purposedly described in broad terms to leave teams room for creativity and avoid the risk that challenges are perceived as additional schoolwork to complete. Challenge 1 (i.e., a test on basic information on what a healthy and sustainable lifestyle means) and 30 (i.e., organize an event at school to show what they have done in the program) are compulsory and teams are free to choose five out of the 30 challenges on the list and have to complete them over the course of the school year. Teams are required to choose at least one challenge for each main program topic (i.e., healthier and more sustainable behaviors, and physical activity) and to complete a total of seven challenges (two compulsory and five elective). Teams that cannot complete the required number of challenges are excluded from the program. Teams are supported by the teachers and program staff by regular (monthly) meetings and over the phone upon request. Students are encouraged to share the products of their work on the program’s and team’s Instagram pages. Publishing contents on social media is supposed to contribute to further dissemination of messages on health and sustainability to peers. After the completion of each challenge, the teams’ output is graded from one to ten by program staff based on creativity, completeness and degree of effort provided. The ranking is updated monthly and shared with teams. The winning team is announced at the final event organized at the end of the school year. In sum, the program exploits some elements of gamification including goal setting (i.e., the challenges), rewards and recognition (i.e., receiving feedback and grades for their outputs), competition with other teams (i.e., the monthly ranking), team cooperation and taking new roles (e.g., some students take leadership in their teams and are assigned formal roles such as team leader, leader of communication or leader of a specific challenge).

**Table 1:** Food Game challenges

Challenge #	Challenge title	Challenge description and examples of teams output (2021–2022 edition)
1*	Knowledge test	Teams were required to watch informative videos to learn the basics of the connection between health, lifestyle and sustainability. Students learnt the basics of the Mediterranean diet. They then completed a knowledge test.
2	Design a team logo promoting healthy diet	Figure S1 displays some examples of logos designed by teams
3	Organize a “Fruit Day” at school	A team organized a fresh fruit distribution as part of a school event that included a three-kilometer walk around the school.
4	Produce educational materials	A team placed real-size cardboard vending machines in their school. These vending machines offered fruit instead of the usual snacks.
5	Organize an intergenerational walk	Not chosen by any team. It consists of organizing a walk with people from a different generation such as older adults or young children.
6	Assess the amount of food waste in their household	Members of a team kept track of their food waste for four consecutive days and posted results on Instagram.
7	Organize a thematic flash mob	A team organized a flash mob with students from a local middle school to promote physical activity. The event addressed visually impaired students.
8	Organize a group of students to go to school on foot	A team organized Thursday group walks with other students from their school. As groups reached the school, they engaged in a 10-minute basketball session.
9	Organize a second “Fruit Day” at school	Same as challenge #3.
10	Prepare traditional healthy food in group	A team designed a full traditional healthy meal to be prepared with their families at home. Recipes were posted on Instagram.
11	Create a presentation to promote the Mediterranean diet	A team created and disseminated on Instagram a PowerPoint presentation on the benefits of the Mediterranean diet.
12	Examine marketing techniques of a food commercial	Not chosen by any team. It consists of analyzing how marketing techniques can be used to promote unhealthy products.
13	Paint a mural at school featuring a health topic	After getting consent from the school administration, a team created a painted mural on a wall of their school (Figure S2).
14	Launch a *mail bomb* on a health topic	Not chosen by any team. It consists of disseminating a health message to as many people as possible by e-mails.
15	Organize a discussion with another team on a health topic	Not chosen by any team. It consists of setting up a public discussion on a health topic with another team in Food Game. The video of the event has to be posted on Instagram.
16	Invent a new challenge	A team produced laundry soap bars from waste frying oil.
17	Go grocery shopping for their family (four consecutive meals)	Not chosen by any team. It consists of going grocery shopping with their families to encourage them to purchase healthy food products to be used in four consecutive meals.
18	Promote environmental-level change at the school level	A team organized “energizing” school breaks that continued throughout the entire school year. During these sessions, students were encouraged to stay active with exercises and music in the school garden.
19	Shoot a cooking video showing the amount of waste produced	Not chosen by any team. It consists of shooting a video of a cooking session to showcase the amount of waste produced and strategies to reduce it.
20	Organize a thematic meeting with primary or middle school children	A team organized a lecture on healthy eating with children from a local primary school. The session included games and active learning. The video was then posted on Instagram.
21	Survey peers on their opinion about the team’s Food Game products and write a report	Not chosen by any team. It consists of surveying peers on their opinion about the team’s Food Game outputs. Students then write a report to describe results.
22	Interview students who participated in Food Game in the past	A team interviewed students from past Food Game editions on their experience with the program and the effects on their knowledge, opinions and habits.
23	Calculate one’s ecological footprint	Students were instructed on how to measure their carbon footprint. They then made a written public commitment to reduce their footprint and that of their family. Results of their efforts were then publicized in school and posted on Instagram.
24	Calculate the amount of packaging waste over the course of four days	Not chosen by any team. A team keeps track of the amount of waste packages they accumulated at school and at home over a week. Results are posted on Instagram
25	Organize a party offering healthy food	A team organized a party in a city park. Healthy food was offered. Peers were invited to join and play different sports.
26	Organize the cleaning of a public green space	It consisted of organizing the cleaning of a public green space such as a park or the school garden. Three teams worked together with a local community association to clean up the school garden.
27	Become environmental tutors for a day	Not chosen by any team. After gaining consent from the shop owner, students spend a day in a in a food shop to help clients read food labels, make healthier purchases and reduce purchase of unnecessary packaging.
28	Learn how to create a garden	After getting consent from the school administration, a team built a vegetable garden in their school with the help of a team member’s grandfather.
29	Organize a packaging free shopping day	Not chosen by any team. It consists of organizing a shopping day with the goal of reducing the amount of packaging as much as possible.
30*	Organize an event at school to give visibility to what the team has done in Food Game	A team recorded videos of their experience with Food Game to be posted on Instagram and shown during the program’s final event at school.

Note. * Compulsory challenge

## Aims

The primary aim of the current process evaluation study was to examine how Food Game worked in practice, understand its mechanisms of change and assess any variation in student outcomes (Moore et al., 2015). Process evaluation is not meant to determine the effectiveness of an intervention but may represent a preliminary step to design an effectiveness evaluation study. Our key research questions were: (a) What are the program’s key components (input, output, expected outcomes) and proposed mechanisms of change? (b) What is the teachers’ and students’ experience with the program and its gamified components? (c) What barriers and facilitators to the implementation of the program are perceived by the staff, teachers and students? (d) Was there any change in students’ behavioral (adherence to the MedDiet and pro-environmental behaviors) and psychosocial antecedents (attitudes and social norms) of healthy dietary choices over the course of the schoolyear?

In this study, we were guided by the principles of Empowerment evaluation (Fetterman & Wandersman, 2005) where evaluation is conducted jointly with program staff and end-users with the aim of improving the program. The outcomes of the evaluation consist of corrective feedback, deeper knowledge on the program and support to a capacity building process among program staff and stakeholders.

## Mixed Method Design

The evaluation design consists of a convergent parallel mixed method study with quantitative and qualitative data collected and analyzed concurrently (Creswell & Plano Clark, 2011). The qualitative study consisted of focus groups with students and interviews to program staff and teachers. The quantitative study consisted of a three-wave longitudinal survey to collect information on students’ behaviors and psychosocial characteristics (i.e., attitudes and social norms) (Godin & Kok, 1996). Considering the lack of a control group, the quantitative study was meant to assess variation in the outcomes with the purpose of informing the study design of a future experimental evaluation study. Data from the two studies was collected during the 2021–2022 school year, was analyzed independently and results were interpreted together. Participants did not receive any incentive for their participation. Both studies were approved by the university’s Institutional Review Board and were conducted in accordance with the Declaration of Helsinki. All participants provided their written informed consent to participate in this study.

## Qualitative Study

### Methods

#### Data Collection

Program’s documents (i.e., description on the Pro.Sa. health promotion intervention repository^2^) were reviewed and staff interviews were conducted to collect the necessary information on the program. Teachers of teams that participated in Food Game past editions were also interviewed to understand their experience with the program. All the student teams from the 2021/2022 edition were invited to participate in focus group interviews. In order to facilitate the setting of a typical focus group, teams were instructed to select a maximum of 8–10 of them to take part in the interview. Focus groups were conducted in school venues or online in March-April 2022. Each focus group was moderated by a researcher with training and expertise in qualitative research. Discussions used a semi-structured script with broad questions followed by open-ended prompts. Teachers were asked to describe their past involvement in Food Game, their motivations to participate, their perception of the strengths of the program and barriers to implementation. The topic guide for the students focus group was organized in three main sections: experience with the program, experience with gamified activities and perceptions of outcomes.

#### Participants

Four staff and five teachers from four different schools were interviewed. Seven focus groups were conducted with a total of 42 students (45.2% female) representing 14.6% of the 288 students who participated in Food Game. Each focus group session lasted approximately one hour and consisted of four to eight participants.

#### Analyses

NVivo was used to support data management and analysis. A thematic analysis of transcripts was conducted (Braun & Clarke, 2006). To guide the analysis of the program’s components, we drew a logic model (Kaplan & Garrett, 2005). The logic model visually represents the program’s core components and expected outcomes. Logic models generally include four major components: inputs (i.e., human, financial and organizational resources), activities intended to bring about the changes or the results, outputs (i.e., products of activities) and expected outcomes. We created descriptive codes of the model components and analyzed all transcripts based on these components.

### Results of Qualitative Study

**Fig. 1:**
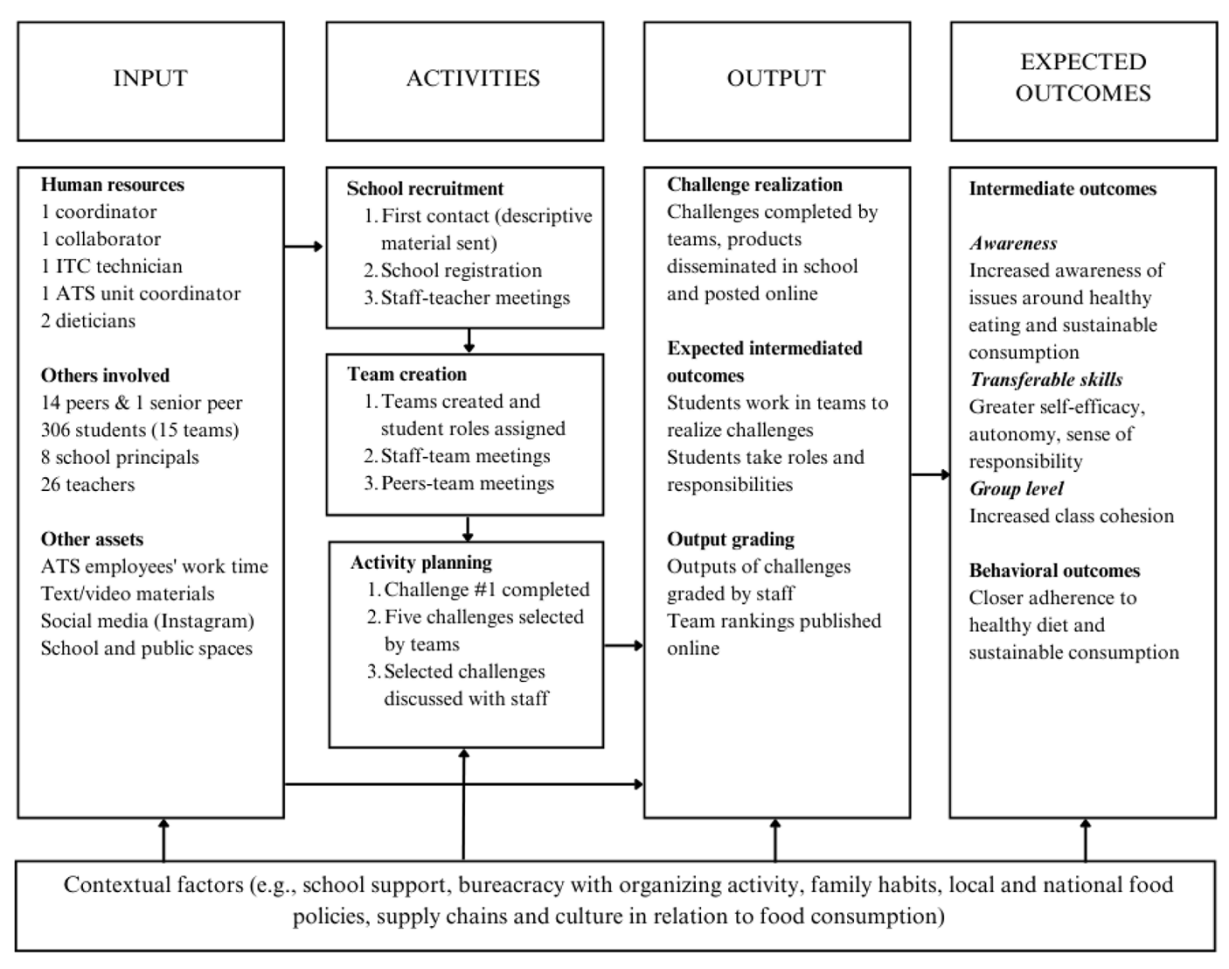
Food Game logic model

Figure 1 displays the Food Game logic model as derived from staff interviews. Results of teachers interviews and students focus groups are organized into topics reflecting participants’ experience with the program and its gamification strategy as well as perceived outcomes on attitudes and behaviors. Quotes representative for each topic are included in the text.

### Teacher Interviews

Teachers reported an overall positive experience with the program. They said they appreciated Food Game because it encouraged group work on issues of interest for students, thus offering them the opportunity to foster their skills in communicating, collaborating with others and work on conflict resolution. Teachers appreciated how students were encouraged to take responsibilities, be autonomous, feel important and capable of doing things, and be creative to complete the challenges. In other words, the teachers’ interest in the program went beyond promoting health and sustainability and what really motivated them was the expectation that the program could help support students in developing transferable skills.

I promote Food Game in my school as some sort of team building. You have students working in groups and groups have to be productive and are constantly compared with other groups. Then obviously there is the program’s educational aspect as they learn things about the importance of healthy eating and things like that. (Teacher, School 1)

What worked the most for us was fostering students’ protagonism. Students worked with great autonomy. They were interested in the program’s topics and, since they are familiar with social networks, it was easy for them to use them as a means of communication for the result of their work. We let them work with little direction from any adult. (Principal, School 4)

Some teachers reported that students’ interest in the program was low at the beginning and participation had to be made mandatory rather than elective. In these cases, group work and result achievement were more difficult. Lack of support from the school management and bureaucracy were mentioned as additional barriers to the program implementation.

There is so much bureaucracy in the school system nowadays. For example, organizing a field trip [as part of the activities to complete a challenge] with students is a nightmare […] if anything bad happens, it is the teacher’s responsibility. […] School principals are not always supportive of these initiatives. (Teacher, School 2)

### Students Focus Groups

#### Experience with the Program

Overall, students reported an interest in the topics addressed under Food Game, though all teams said they had not freely chosen to join the program but rather had been encouraged by their teachers. Students described two key aspects as particularly enjoyable: being able to engage in practical activities where they could use their creativity and their prior knowledge; and being free to choose which topic to address and self-organize to put their idea into practice.

“It’s been nice to see how our class worked together as a team, how each one of us put forward ideas […]. [Participating in Food Game] also strengthened our class as a group. We felt free in this project because there was really no right or wrong. Every idea was put into practice and everyone felt they had contributed somehow.” (Team #4).

Some participants reported how Food Game changed group relations in their class. Group work encouraged social interaction and increased their sense of belonging and group cohesion.

“I was in charge of publishing our work on Instagram. What happened in our team is that I had to reach out to some classmates of mine I had barely ever talked with before. Suddenly, we found ourselves doing things together and chatting for the first time.” (Team #10).

#### Experience with Gamification

Students said they had fun in Food Game. Even though some groups described conflict or organizational difficulties related to decision-making and lack of interest among some members, collaboration among teams was highlighted as a key driver to program appreciation:

“What matters to us is that we have fun together. No drama if we don’t place first in a challenge.” (Team #7).

Most teams described competition with other teams as a motivator to do their best and achieve better results and products. Students reported that they often looked at their team’s ranking: knowing that their work would be graded encouraged teams to put greater effort into any challenge.

“[The team ranking] encouraged us to give our best and do things better. It’s also a way to interact with other teams in other schools.” (Team #9).

“Not competing would make Food Game too much of a game and this means you could easily underestimate the work involved, thus not putting the greatest effort into each challenge.” (Team #10).

Remarkable exceptions to the narrative around the role of inter-team competition was represented by two teams that struggled in organizing their work, reported poor group climate and eventually ranked low in the competition.

“Group cohesion was low. We didn’t manage to organize much and couldn’t really identify with our team.” (Team #15).

#### Perceptions of Outcomes

Most teams described Food Game as a fun way to learn new things, including transferable skills, and put them into practice. Changes referred to individual study participants but also to their peer. Some students reported changes in habits, greater knowledge and more positive attitudes towards healthy eating and sustainable lifestyles.

“Food Game changed the way I eat. In the past, I would often skip meals and breakfast in particular. Now, I’m more aware that skipping meals is unhealthy and I do it less often.” (Team #9).

“I realized that before Food Game I didn’t pay much attention to the nutritional and ecological characteristics of the food I ate. Now, I do realize what those things mean and notice them.” (Team #7).

“In a challenge, we cooked healthy dishes together. It was fun and I think that many people who thought that healthy eating was boring changed their mind.” (Team #4).

Some students said they had noticed some changes in their peers because they either looked more proactive or discussed about these issues after looking at the products of challenges at school or on social media.

*“As regards my friends, I think [Food Game] may have had some effect. My friends and I are taking our bikes more often and because we did that in Food Game we post our rides on social media […] I’m now involving more people who are not just my closest friends.” (Team #4)*

*“Social networks help disseminate any message among people our age. I was glad to see that people our age are open to these [sustainability] issues. I thought they didn’t care. It makes a difference for the 20 people of our class, but we can talk about these things with others in our school or at home.” (Team #11)*

## Quantitative Study

### Methods

#### Data Collection

A three-wave longitudinal design was conducted with sampling undertaken at T_1_ (before the program in November 2021), T_2_ (three months into the program in February 2022) and T_3_ (at the end of the school year in May-June 2022). All students whose parents had signed the informed consent to participate in the study were eligible and were sent a link to the three surveys by email. The behavioral outcome variables (adherence to the MedDiet) and psychosocial antecedents (attitudes and perceived social norms about the Mediterranean diet and sustainable lifestyles) were measured at each wave, whereas students’ experience of gamification was measured only at T_3_. The survey was co-developed with program staff and pilot tested for length and clarity with three non-participating adolescents. Additional data on the program implementation was collected.

#### Measures

Participants completed items on socio-demographic characteristics (e.g., gender, age) and experience in Food Game (e.g., amount of time spent working on activities related to the program). We used the validated Italian version of each measure when available. In any other case, prompts and items in English were translated (and back translated for accuracy) into Italian by native speakers. To ensure the translated version maintained the original meaning, any incongruence between each back translated English version and the original one was resolved through discussion.

#### Mediterranean Diet Adherence

We used a revised version of the Mediterranean Diet Index (Benedetti et al., 2016). This index was developed in Italian and is a composite score that summarizes the frequency of consumption of 12 types of food (e.g., pasta, pulses, vegetables), and the type of oils/fats used for cooking (e.g., extra virgin olive oil). Respondents were prompted to think about the previous month and to indicate the frequency they had each type of food. A score ranging from 0 to 4 (Table S1) was assigned to the frequency of consumption of each food category according to the degree of adherence to the principles of the MedDiet. As for the use of cooking oils and/or fats, four points were assigned to frequently using of extra virgin olive oil and avoiding other oils, butter and margarine. The adherence indicator could assume values between 0 and 64 with greater scores indicating greater adherence. Food some categories were slightly revised by Food Game nutritionists compared to the original version in order to avoid overlaps and better reflect the most recent guidelines on the MedDiet.

#### Attitudes and Perceived Social Norms About Healthy Eating

We used a set of three items adapted from de Leeuw et al. (2015). Respondents were asked to rate the extent to which they considered healthy eating useful, pleasant and nice. Perceived social norms were operationalized as injunctive norms (i.e., perception of others’ approval of healthy eating). Because the program aims to foster peer-to-peer learning, peers were used as reference group. In both cases, answers were scored from 1 (not at all) to 5 (very much).

#### Pro-environmental Behaviors

Selected items from the Italian validation of the Pro-Environmental Behaviors Scale (Menardo et al., 2020) were used. After excluding items deemed not appropriate for the target group of adolescents (e.g., dishwasher use), three items were selected to assess behaviors indicating interest in the environment: “How frequently do you watch television programs, movies or internet videos about environmental issues?”, “How often do you talk to family members about their environmental behavior?”, and “How often do you talk to your peers about their environmental behavior?”. The answers were scored from 1 (never) to 5 (always). The scale also includes two additional dichotomous items (0 = no; 1 = yes): “Are you currently a member of any environmental or wildlife protection group?” and “During the past year have you contributed money to environmental, conservation or wildlife protection?”.

#### Gamification

In order to assess different dimensions of the students’ experience with gamification, three subscales of the Gameful Experience Scale (GAMEX) were used (Eppmann et al., 2018): Enjoy (six items; e.g., “Playing [Food Game] was fun”), Creative thinking (four items; e.g., “Playing [Food Game] sparked my imagination”) and absence of negative affect (three items; reversed score; e.g., “While playing the game I felt upset”). Answers were scored from 1 (strongly disagree) to 7 (strongly agree).

#### Data Analyses

Data was analyzed using multi-level regression models in MLwiN 2.33 (Rasbash et al., 2009). Random intercepts were incorporated into the modelling framework to account for the hierarchical structure in the data. Level one random effects were at the within participant level (T_1_, T_2_, and T_3_ repeated measures), nested within individual-specific level two random effects, nested within level three (school classes) so allowing the inclusion of variance due to different classes. Two models were tested. First, a random intercept model tested the significance of variation in students’ outcomes at the three levels. Then, changes in the outcome variable over time were modelled indicating time of measurement (T_1_ = 0, T_2_ = 1, and T_3_ = 2) as a predictor with T_1_ as the reference category. Gender and age were included as control variables.

### Results of Quantitative Study

All teams completed the minimum number of challenges required (7). Teams completed a total of 19 challenges on healthier dietary choices (*M* = 2.07; *SD* = 0.59; range 1–3) and 15 on sustainable behaviors (*M* = 1.53; *SD* = 0.64; range 1–3).

**Fig. 2:**
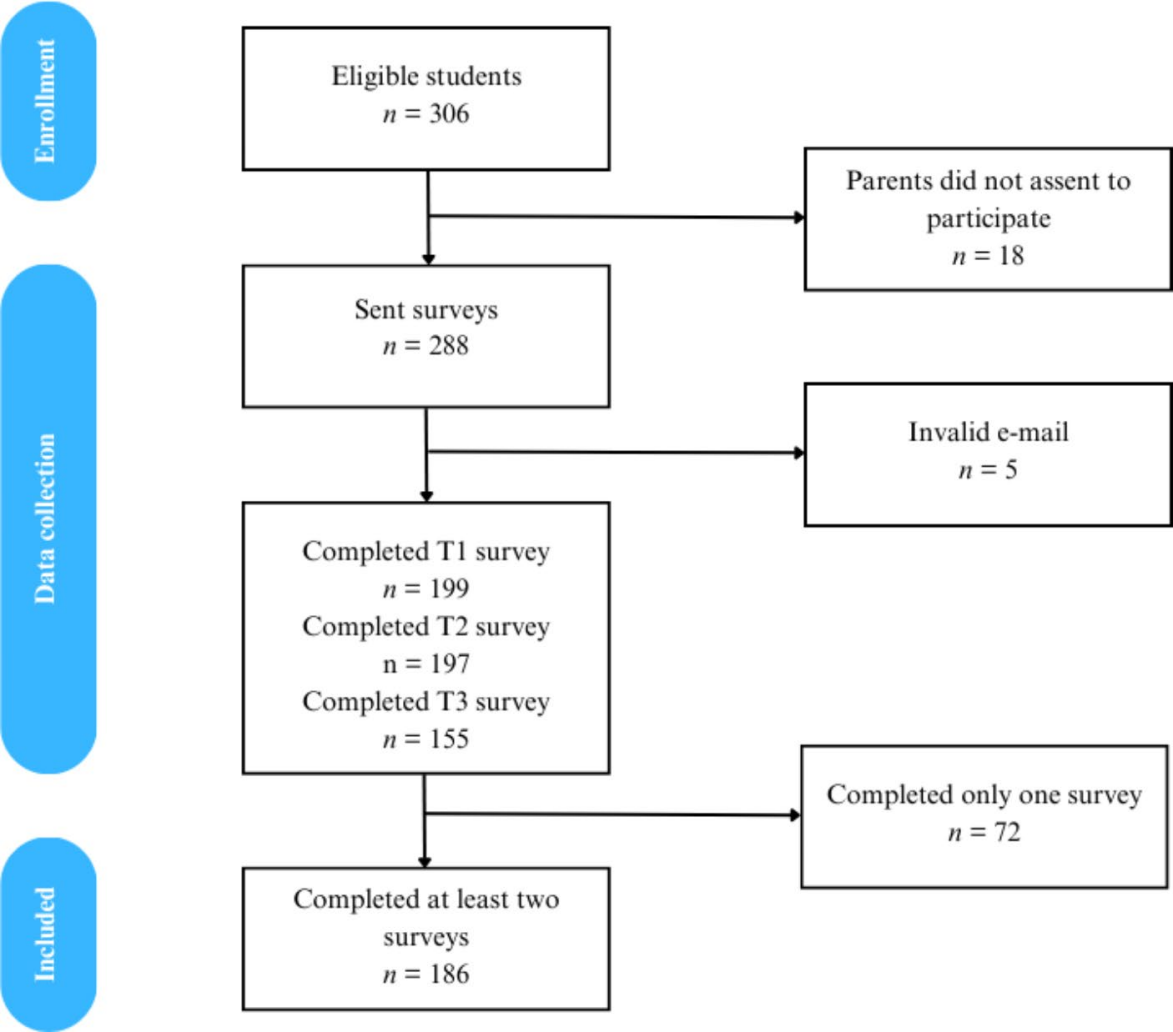
CONSORT flow diagram for enrolment and retention in longitudinal study

Figure 2 illustrates the number of students retained and excluded at each step of data collection. Those who participated in at least two waves were included in the analytical sample (N = 186). Table 2 displays participants’ socio-demographic characteristics and information on their engagement with the program.

**Table 2:** Participants’ socio-demographic characteristics and engagement with the Program

	Sample *(N = 186)*
**Socio-demographic characteristic**	
Age in years, mean (SD)	15.6 (0.638)
Gender (female)	95 (51.1)
Migrant background (yes)	10 (5.4)
**Engagement with Food Game**	
Formal role in own team (yes)	75 (40.3)
N. of hours per month spent on the program	
Up to five	32 (17.2)
Between six and ten	71 (38.2)
More than ten	38 (20.5)

The measure of attitudes towards healthy eating showed acceptable internal consistency at all waves (T_1_*α* = 0.775; T_2_*α* = 0.806; T_3_*α* = 0.812). The measure of pro-environmental behavior showed acceptable internal consistency at all waves (T_1_*α* = 0.707; T_2_*α* = 0.789; T_3_*α* = 0.821). The maximum score possible was seven. Mean scores of gamification dimensions were 4.42 (*SD* = 1.63) for enjoyment, 4.33 (*SD* = 1.74) for creative thinking and 2.89 (*SD* = 1.77) for reversed scores of absence of negative affect. This shows that participants generally had fun in the program, found it stimulating and did not experience emotional discomfort in taking part in the activities. All three scales showed good internal consistency (enjoyment *α* = 0.964; creative thinking *α* = 0.946; absence of negative affect *α* = 0.865).

### Changes in Outcome Variables

Table 3 shows results of multilevel analyses on outcome variables. In Model 1, the variance of outcome variables at Level 3 (classes) was not statistically significant and was therefore removed from all subsequent analyses. Results of Model 2 show predicted mean scores of MedDiet adherence did not vary across waves and were 42.96 (*SD* = 4.654) at T_1_, 43.44 (*SD* = 4.574) at T_2_ and 42.839 (*SD* = 4.819) at T_3_. Engagement in pro-environmental behaviors increased from T_1_ (2.07, *SD* = 0.563) to T_2_ (2.25, *SD* = 0.561) but returned to slightly above pre-intervention levels at T_3_ (2.17, *SD* = 0.598). The proportion of students participating in environmental or wildlife protection groups increased from 4.1% at T_1_ to 6.2% at T_3_. Similarly, there was a 60% increase from 5.3% at T_1_ to 13.5% at T_3_ in the proportion of students who reported having donated money to such organizations. Statistically significant changes occurred in psychosocial antecedents of healthy dietary choices. Predicted mean scores of attitudes towards healthy eating remained stable from T_1_ (3.53, *SD* = 0.512) to T_2_ (3.60, *SD* = 0.515) but increased at T_3_ (3.84, *SD* = 0.518). Predicted mean scores of perceived peer approval towards healthy eating were significantly greater at T_2_ (3.73, *SD* = 0.505) and T_3_ (3.81, *SD* = 0.522) compared to T_1_ (3.51, *SD* = 0.512).

**Table 3:** Multilevel regression models predicting change in outcomes at the three time points

	*MedDiet adherence*		*Attitudes healthy eating*		*Social norms healthy eating*		*Pro-environment behaviors*	
	*B*	*[95% CI]*	*B*	*[95% CI]*	*B*	*[95% CI]*	*B*	*[95% CI]*
**Fixed effects**								
Constant	44.009***	[42.565, 45.454]	3.553***	[3.381, 3.726]	3.557	[3.332, 3.782]	2.175	[1.997, 2.353]
Time [T2]	0.294	[-0.636, 1.225]	0.070	[-0.047, 0.187]	0.214*	[0.026, 0.401]	0.157**	[0.040, 0.274]
Time [T3]	-0.322	[-1.334, 0.690]	0.248***	[0.121, 0.375]	0.280**	[0.078, 0.481]	0.081	[-0.045, 0.208]
Gender [Male = 1]	-1.724	[-3.505, 0.057]	-0.038	[-0.250, 0.174]	-0.101	[-0.365, 0.164]	-0.216	[-0.435, 0.003]
Gender [Undisclosed = 1]	-1.897	[-5.66, 1.866]	0.027	[-0.420, 0.474]	0.111	[-0.447, 0.699]	0.262	[-0.200, 0.725]
Age^a^	-1.695**	[-2.958, -0.431]	0.042	[-0.108, 0.193]	0.023	[-0.447, 0.669]	0.132	[-0.023, 0.287]
**Random effects**								
Level: Class^b^	3.565		0.006		0.028		0.063	
N	14		14		14		14	
Level: Participant^c^	25.022***		0.343***		0.426*		0.373***	
N	169		169		169		169	
Level: Time^c^	18.015***		0.283***		0.727		0.283***	
N	449		447		447		447	

Note. MedDiet = Mediterranean diet; CI = confidence intervals; Effects are reported as unstandardized regression coefficients; ^a^ The predictors are centered at the grand mean; ^b^ Random effect removed; ^c^ Results of two-level models are reported *** *p* < .001 ** *p* < .01 * *p* < .05.

## Discussion

This mixed method process evaluation examined Food Game, a gamified school-based program promoting healthier and more sustainable behaviors among high school students in an urban area in the North of Italy. The program consists of a competition where teams of students are asked to perform peer-led activities in groups aimed at promoting awareness of environmental issues related to lifestyle and dietary patterns. The MedDiet is proposed as a model of healthy and sustainable dietary pattern reflecting the traditional Italian cultural model (Benedetti et al., 2016; Dernini & Berry, 2015). A logic model was devised to visually represent the program’s key components organized into inputs (i.e., human resources), activities intended to bring about the changes or the results, outputs (i.e., products of activities) and expected outcomes (Kaplan & Garrett, 2005). Interestingly, the overall architecture of the program attracted teachers’ attention beyond their interest in having students work on sustainability issues. The alignment of the program with schools’ pedagogical mission is a strength as it facilitates teachers’ buy in and adoption on scale in the future. Students, however, were not always interested in participating in the program in the first place. Future studies should assess whether interest increased over time and what effects variations in interest and motivation had on student engagement and the program’s outcomes.

What is truly innovative from an implementation perspective, however, is the use of gamification as the key motivational design to keep students engaged, foster group collaboration and increase the quality of their work (Hamari & Koivisto, 2015). The adoption of gamification components in offline school-based interventions is relatively rare compared to digital health interventions (Edwards et al., 2016; Johnson et al., 2016). Our analyses suggest that, overall, the program’s gamification design was successful. Both survey and focus group data indicate that taking part in Food Game was enjoyable and engaging for students who reported high levels of commitment. Students reported that the game stimulated the expression of their creativity through self-organized group work and collaboration on practical activities. Importantly, for most students competition with other teams was described as a motivating factor to work harder and achieve better results and products rather than representing a source of stress. Students’ accounts seem to disconfirm studies conducted in formal education contexts and indicating that some typical elements of gamification, such as competition and rewards, may lead to unintended consequences. A competitive context may stimulate increased effort but can also be detrimental to fairness and respectful attitudes towards the opponents or harm the losing counterparts’ motivation and performance. Similarly, receiving rewards may discourage students and increase extrinsic motivation at the expense of intrinsic motivation (see, for example, Domínguez et al., 2013; Hanus and Fox, 2015). Future studies could further examine the applicability as well as the potential unintended effects of the gamification approach in this program and in other similar objectives of non-formal education.

Our analyses showed no significant change in students’ dietary choices (i.e., adherence to the MedDiet) and only temporary changes in behaviors demonstrating an interest in the environment. Other indicators of pro-environmental behaviors, such as participating or donating money to environmental or wildlife protection groups, did show some increases at the end of the school year. Some changes in the expected direction were observed in the two measured psychosocial antecedents of healthy dietary choices (i.e., favorable attitudes and perceived peer approval towards healthy eating).

These effects are somehow promising at this stage and, despite having been achieved in an uncontrolled study, allow to draw some preliminary conclusions and to provide suggestions for improvement.

First, the goal of changing the students’ dietary and sustainability choices in just one school year may have been too optimistic given the relatively low intensity of the program. All the student teams received training on the program’s target topics (i.e., Challenge #1) and spent a variable, in some cases limited, amount of time to complete the challenges on healthier diet and related sustainability issues. This was apparently enough to affect students’ attitudes and normative perceptions, although it can be argued that it may not represent a sufficient ‘dosage’ (i.e., the amount of intervention received) to achieve any significant behavioral change (Rolfe et al., 2009). Therefore, possible avenues for strengthening the program may include increasing dosage by prolonging its duration to two school years and/or focusing on a single outcome (e.g., healthy diet).

Second, the lack of effects on behavioral outcomes may also be explained from a socioecological perspective. Indeed, it is difficult to achieve any significant change given the complex array of factors in the different socio-ecological systems that determine the diet and lifestyle in general (Stokols, 1996). These include family dynamics and food habits, environmental factors at school and community level, characteristics of local and national food policies, supply chains and overall culture (Raza et al., 2020). None of these factors were addressed by Food Game in its current version but some potentially could in the future. For example, the program could be revised so that it can encourage students to engage in advocacy and other activities to promote change in school and community aspects that do affect their lifestyle choices, for instance school healthy eating policy, offer of snack vending machines, and food retailers around the school (Townsend & Foster, 2013). The literature on youth participation in prevention offers methodological guidance for young people to contribute to policy reform at the local level (Warren & Marciano, 2018).

### Limitations

This evaluation has a number of boundary conditions that could be expanded in further studies. First, the study design did not allow to measure the causal impact of the program. Second, given this study was the first to examine this novel program, we were not able to assess important aspects of the implementation including fidelity, dose and reach (Grant et al., 2013). Third, due to the limited resources, we were not able to follow up with students who dropped out or did not complete any survey. The reasons for a relatively high dropout rate, especially at T_3_, are therefore not clear at this stage. Future studies may use different strategies to collect data (e.g., in-class administration) to better measure completion rates and systematically document reasons for non-completion. Fourth, in this study, multilevel models were used to account for variation at different levels, though the limited sample size did not allow to investigate the school effect on students’ behavior (Townsend & Foster, 2013). Fifth, in this study the students’ socio-economic status was not taken into account as a control variable. This is a limitation because there is evidence that adherence to the MedDiet and healthy dietary patterns in general is greater among socioeconomic subpopulations of higher status (Delbosq et al., 2022; Iaccarino Idelson et al., 2017). Future studies should consider social inequalities as a determinant of lifestyle patterns.

## Conclusions

Despite research limitations and the need for further examination, the findings of this study provide preliminary support for Food Game as an acceptable and engaging gamified intervention though there is yet not sufficient evidence that it is also promotes healthier and more sustainable behaviors. Results contribute to the limited evidence base for offline gamified school-based interventions and offer insight into the applicability of the gamified approach to foster interest and sustain long-term engagement in preventive programs in other settings and on other health issues.

### Electronic Supplementary Material

Below is the link to the electronic supplementary material.

**Supplementary Material 1: Figure S1**. Examples of logos designed by teams to complete challenge #2

**Supplementary Material 2: Figure S2**. Two x two-meter-wide painted mural created by a team to complete challenge #13

**Supplementary Material 3: Table S1**. Scores of the Mediterranean diet index revised

## Data Availability

Data supporting the findings of this study can be obtained from the corresponding author, GA, upon reasonable request.

## Footnotes

1 Food Game also aims to increase students’ physical activity but this aspect was not considered as it was beyond the scope of this study.

2 https://www.retepromozionesalute.it/bd2_scheda.php?idpr2=5242.
