# Supplementary figures and images for: Process Evaluation of Food Game: A Gamified School-Based Intervention to Promote Healthier and More Sustainable Dietary Choices
Source: J Prev (2022). 2023 Aug 6;44(6):705–27. doi: 10.1007/s10935-023-00741-3 (PMC10638118; doi:10.1007/s10935-023-00741-3)

## Figure S1. Examples of logos designed by teams to complete challenge #2.


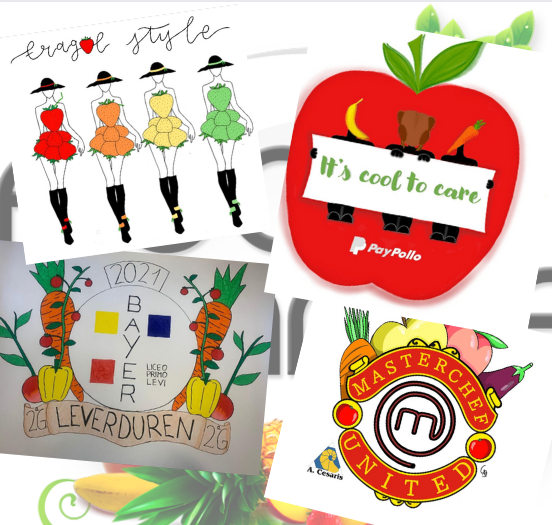

Supplement: Supplementary file 1 — Supplementary Material 1: Figure S1. Examples of logos designed by teams to complete challenge #2 [file 10935_2023_741_MOESM1_ESM.docx]

## Figure S2. Two x two-meter-wide painted mural created by a team to complete challenge #13.


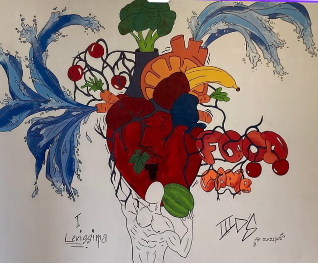

Supplement: Supplementary file 2 — Supplementary Material 2: Figure S2. Two x two-meter-wide painted mural created by a team to complete challenge #13 [file 10935_2023_741_MOESM2_ESM.docx]
