# Supplementary material for: Process Evaluation of Food Game: A Gamified School-Based Intervention to Promote Healthier and More Sustainable Dietary Choices
Source: J Prev (2022). 2023 Aug 6;44(6):705–27. doi: 10.1007/s10935-023-00741-3 (PMC10638118; doi:10.1007/s10935-023-00741-3)
Supplement: Supplementary file 3 — Supplementary Material 3: Table S1. Scores of the Mediterranean diet index revised [file 10935_2023_741_MOESM3_ESM.docx]

Table S1.

Scores of the Mediterranean diet index revised

| Food | More than once a day | Once a day | A few times per week | Less than once a week | Never |
| --- | --- | --- | --- | --- | --- |
| Pasta, rice and bread | 4 | 3 | 2 | 1 | 0 |
| Vegetables* | 4 | 3 | 2 | 1 | 0 |
| Fruit | 4 | 3 | 2 | 1 | 0 |
| Milk | 3 | 4 | 2 | 1 | 0 |
| Dairy products and cheese | 1 | 1 | 4 | 3 | 0 |
| Fish | 1 | 3 | 4 | 2 | 0 |
| White meat (poultry) | 0 | 1 | 4 | 3 | 2 |
| Red meat (beef, pork, lamb)* | 0 | 0 | 2 | 4 | 3 |
| Processed meat (e.g., salami) | 0 | 0 | 1 | 3 | 4 |
| Eggs (also if used in preparations like cakes) | 0 | 2 | 4 | 3 | 1 |
| Dried fruit (e.g., almond, nuts) and seeds (e.g., sunflower seeds* | 4 | 3 | 2 | 1 | 0 |
| Pulses (e.g., beans, lentils)* | 3 | 4 | 2 | 1 | 0 |
| Note. * Item differs from Benedetti et al. (2016) index version. | | | | | |
